# Lipid Transport and Metabolism at the Blood-Brain Interface: Implications in Health and Disease

**DOI:** 10.3389/fphys.2021.645646

**Published:** 2021-03-30

**Authors:** Fabien Pifferi, Benoit Laurent, Mélanie Plourde

**Affiliations:** ^1^UMR CNRS MNHN 7179 MECADEV, Brunoy, France; ^2^Département de Biochimie et de Génomique Fonctionnelle, Université de Sherbrooke, Sherbrooke, QC, Canada; ^3^Centre de Recherche sur le Vieillissement, CIUSSS de l’Estrie – CHUS, Sherbrooke, QC, Canada; ^4^Département de Médecine, Université de Sherbrooke, Sherbrooke, QC, Canada; ^5^Institut sur la Nutrition et les Aliments Fonctionnels, Université Laval, Quebec City, QC, Canada

**Keywords:** n-3 PUFA, blood-brain-barrier, blood-cerebrospinal fluid barrier, cholesterol, lipid transport

## Abstract

Many prospective studies have shown that a diet enriched in omega-3 polyunsaturated fatty acids (n-3 PUFAs) can improve cognitive function during normal aging and prevent the development of neurocognitive diseases. However, researchers have not elucidated how n-3 PUFAs are transferred from the blood to the brain or how they relate to cognitive scores. Transport into and out of the central nervous system depends on two main sets of barriers: the blood-brain barrier (BBB) between peripheral blood and brain tissue and the blood-cerebrospinal fluid (CSF) barrier (BCSFB) between the blood and the CSF. In this review, the current knowledge of how lipids cross these barriers to reach the CNS is presented and discussed. Implications of these processes in health and disease, particularly during aging and neurodegenerative diseases, are also addressed. An assessment provided here is that the current knowledge of how lipids cross these barriers in humans is limited, which hence potentially restrains our capacity to intervene in and prevent neurodegenerative diseases.

## Introduction

The brain is rich in lipids since it contains 24% phospholipids (PLs) and 22% cholesterol by dry matter (Svennerholm et al., 1997). However, the brain has a poor capacity to synthesize lipids which must be supplied from peripheral blood circulation and cross the barriers protecting the entrance of toxic molecules within the central nervous system (CNS) (Igarashi et al., 2007; Lacombe et al., 2018). The liver is the major site of lipids production where the long chain polyunsaturated fatty acid (PUFA) are synthetized. Alpha-linolenic acid (ALA) and linoleic (LA) are designated essential fatty acids because they cannot be synthesized *de novo* and must be obtained from the diet (Bourre, 2006). In the liver, the precursors ALA and LA acids are converted into deocosahexaenoic acid (DHA) and arachidonic acid (ARA), respectively (Scott and Bazan, 1989), but this process is not efficient with less than 1% of ALA and LA converted (Plourde and Cunnane, 2007). In addition to long chain PUFA synthesized in the liver, shorter chains precursors such as ALA are provided by the plasma. These PUFAs are additional lipid sources for the brain. Therefore, long-chain PUFAs must be supplied by the peripheral blood circulation and cross the barriers protecting against the entrance of toxic molecules into the central nervous system (CNS) (Igarashi et al., 2007; Lacombe et al., 2018). A certain level of ALA and LA are thought to be converted into DHA and ARA within the central nervous system (Spector, 2001; Qi et al., 2002). However, the efficiency of this process is unknown and is thought to be insufficient to fulfill the brain requirement for long-chain PUFAs.

The brain has three main blood-brain interfaces: the blood-brain barrier (BBB), the blood-cerebrospinal fluid barrier (BCSFB), and the blood-arachnoid barrier (BAB) (Strazielle and Ghersi-Egea, 2013). Although its role in CNS homeostasis is now well recognized (Holman et al., 2010; Dias et al., 2019), the BAB is avascular and has a smaller exchange area than the BBB and BCSFB (Gomez-Zepeda et al., 2020). Moreover, to our knowledge, the contribution of the BAB to lipid transport and metabolism is unclear and not very well documented. Therefore, this review will focus on the BBB, which is located at the endothelium of the brain microvessels, and the BCSFB, which is located at the epithelium of the choroid plexuses (CPs). The exchange of substances between the blood and the brain is regulated by various mechanisms, including the low permeability of the barrier to most substances and the selective transport of nutrients, ions, peptides, drugs, and hormones using transporters (Zlokovic, 2011; Banks, 2016; Galea and Perry, 2018). The BBB is not only composed of brain microvascular endothelial cells sealed together by tight junctions but can also be extended to the neurovascular unit, which is formed by the basement membrane and neighboring pericytes, astrocytes, neurons, and microglia (Iadecola, 2017). The CP is a tissue found in each brain ventricle and consists of a network of capillaries and epithelial cells. CP capillaries are fenestrated and highly permeable, while cerebral capillaries are highly impermeable. CP epithelial cells (CPECs) surrounding fenestrated capillaries filter water and other substances from the blood and transport them through the epithelial layer into brain ventricles (Lun et al., 2015b). This clear fluid produced by the CP is the cerebrospinal fluid (CSF), which fills the ventricles, cisterns and sulci of the brain as well as the central canal of the spinal cord. In humans, the CP of a young adult produces approximately 500 mL of CSF per day, but only 150 mL is present inside the brain and spinal cord as the CSF is constantly reabsorbed (Damkier et al., 2013). The CP is an evolutionarily conserved structure that is present from lower vertebrates to humans.

Tight junctions link CPECs together to form the CP epithelium, which represents the BCSFB. Although the BBB and BCSFB share the same overall structure, there are significant differences in their permeability. BBB junctions are extremely tight, as they protect the immediate neuronal environment, whereas BCSFB junctions are more permeable and allow the slow leakage of plasma proteins into the CSF. Although there are strong differences in permeability between the BCSFB and BBB, they are both vulnerable to pathophysiological modifications. For instance, defective cholesterol and fatty acid homeostasis in the brain is associated with age-related diseases, especially neurodegenerative diseases (Dietschy and Turley, 2001). Therefore, a defect in these processes will likely modify brain lipid homeostasis. Hence, because lipids are a major component of brain membranes, it is important to understand how they are transported from the blood to the CNS and how modifications in the BBB and BCSFB fatty acid profiles might change their permeability to lipids.

## Lipid Transport Across the Blood-Brain Barrier (BBB)

**FIGURE 1:**
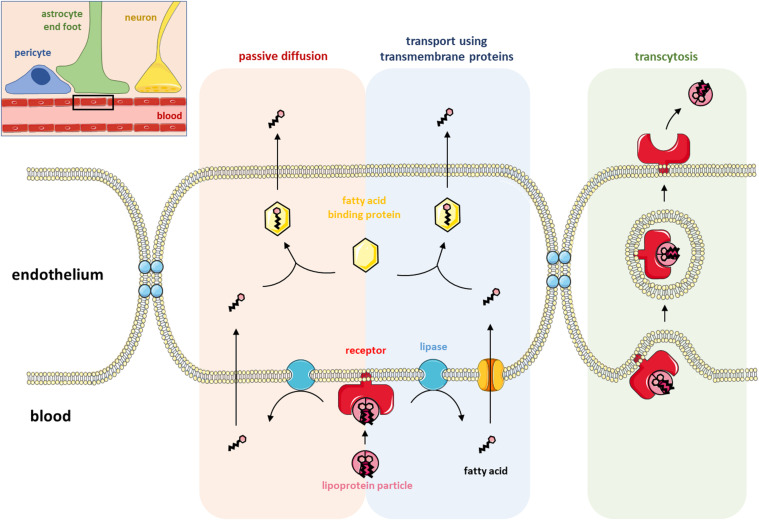
Lipid transport at the blood-brain barrier. The top right corner shows a schematic representation of the blood-brain barrier (BBB) with the blood capillary, a pericyte, an astrocyte end-foot and a neuron. The rest of the figure (a magnified view of the black rectangle) illustrates the three ways lipids can cross the BBB: passive diffusion, transport using transmembrane proteins and transcytosis. Fatty acids (FAs) are locally produced in the endothelium after the hydrolysis of lipoproteins by membrane-associated lipase enzymes. FAs can either passively diffuse across the endothelial membrane or be transferred inside the endothelium by the activity of a transmembrane protein. Once in the endothelium of the BBB, FAs shuttle through the cytosol by binding to FA-binding proteins before being transported into the brain. Lipoproteins can also be directly transported into the brain via receptor-mediated transcytosis involving either caveolin- or clathrin-coated vesicles.

In mammals, brain neurons are highly enriched in cholesterol and in the long-chain PUFAs DHA and ARA. The next section will describe in more details how PUFAs and cholesterol cross the BBB, and a schematic representation of these passages is shown in Figure 1.

### Transport of PUFAs Across the BBB

#### Passive Diffusion

For a long time, the passive diffusion of albumin-associated free fatty acids was thought to be the main form of lipid transport across the BBB (Dhopeshwarkar and Mead, 1973). Transfer via passive diffusion through the membranes of endothelial cells requires the dissociation of the non-esterified fatty acid from albumin and takes place in three steps that do not involve binding to proteins or receptors, namely, adsorption, transmembrane movement, and desorption (Kamp and Hamilton, 2006; Hamilton and Brunaldi, 2007; Lacombe et al., 2018). According to Strosznajder et al., 1996, the permeability of the BBB for an FA depends on three main factors: (1) the relative affinity of the FA for albumin circulating in the bloodstream, (2) the rate of dissociation between the FA and albumin, and (3) the metabolic flow and FA utilization by endothelial cells and nerve cells. Using FAs labeled with a radioactive isotope, these authors demonstrated that ARA diffuses more rapidly through the BBB than palmitic acid (16:0), which itself diffuses more rapidly than DHA (Alberghina et al., 1993, 1994; Strosznajder et al., 1996). The authors also suggested that the slower transport of DHA across the BBB than palmitic acid and ARA potentially limits the passage of DHA from the brain to the blood, hence contributing to the retention of DHA within the CNS. Hence, this mechanism might also participate in the selective retention of DHA within the membrane composition of nervous system cells. Interestingly, these studies also suggested that FA transport through the BBB is not affected during aging (Alberghina et al., 1993, 1994; Strosznajder et al., 1996). Other studies using *in situ* brain perfusion in which radioactive DHA was infused directly into one of the brain carotids reported that less than 10% of radioactive DHA remained in the endothelial cells of the brain vasculature, indicating that DHA crossed the BBB (Ouellet et al., 2009). The binding of DHA to albumin reduced the passage of DHA through the BBB. Since the brain transport coefficient was not saturable, it was suggested that DHA crosses the BBB by simple diffusion (Ouellet et al., 2009). These authors also showed that providing a long-term high DHA diet reduced the brain transport coefficient by 20% (Ouellet et al., 2009). Interestingly, the brain transport coefficient of eicosapentaenoic acid (EPA) was similar to that of DHA, even though brain membranes do not contain EPA.

#### Transcytosis

The second mode of transport of lipids across the BBB is the transcytosis pathway. Two mechanisms of transcytosis involving LDL receptors have been described: clathrin-dependent and caveolae-mediated endocytosis. Clathrin-dependent endocytosis is based on the existence of vesicles coated with clathrin. The assembly of this protein into a structure called a clathrin coat allows the formation of endocytosis pits (Smith et al., 1998). These structures, associated with adapter proteins, can recognize specific sequences of transmembrane proteins and internalize them. Clathrin-dependent endocytosis directs LDL to lysosomes that degrade them to release cholesterol and FAs, while receptors are recycled to the plasma membrane (Kirchhausen et al., 2005; Robinet et al., 2006). Caveolae are membrane vesicles whose formation depends directly on the presence of cholesterol within the membranes (the lipid composition of caveolae is similar to that of lipid rafts). These vesicles contain a transmembrane protein necessary for their formation, caveolin, which has the property of binding cholesterol (Rothberg et al., 1992; Yang et al., 2020). LDL receptors have been observed in these structures at the level of BBB endothelial cells (Dehouck et al., 1997). They can transfer LDLs across the plasma membrane, preventing their degradation. Coculture of endothelial cells with astrocytes showed that the expression of LDL receptors on the surface of endothelial cells was stimulated by the release of a soluble astrocytic factor (Dehouck et al., 1994, 1997).

#### Transport Across the BBB Requiring a Transport Protein

FAs can also cross the BBB when they are esterified to a glycerol backbone such as lysophosphatidylcholine (lysoPC), which has been shown to be the most efficient form of passage through the BBB (Brossard et al., 1997; Bernoud et al., 1998; Lacombe et al., 2018). In addition to the abovementioned mechanisms, Edmond (2001) proposed a model for the specific transport of PUFAs. In the model, lipoprotein receptors are located at the luminal membrane of endothelial cells and are thus not in direct contact with the brain parenchyma. Edmond hypothesized that lipoprotein receptors of the luminal membrane allow the internalization of lipoproteins into endothelial cells. The endothelial cells then process the lipids of the lipoproteins, releasing the PUFAs, which are then selectively transferred to their specific transporters at the abluminal membrane of the endothelial cells. There are a number of candidate transporters at the abluminal side of the membrane that include but are not limited to monocarboxylic acid transporters (MCTs) and/or FA transport proteins (FATPs). Because of the structural variety of PUFAs, there are different FATPs allowing selective transfer across the abluminal membrane (Edmond, 2001). More recent work demonstrated that FATP-1 and FATP-4 are the predominant FA transport proteins expressed at the BBB interface, while FA translocase/CD36 also appears to play a prominent role in transporting FAs across human brain endothelial cells (Mitchell et al., 2011). In 2014, the identification of a member of the major facilitator superfamily, Mfsd2a, as the major transporter for DHA uptake into the brain shifted our view of how PUFAs are transported across the BBB (Nguyen et al., 2014). Mfsd2a is expressed in the endothelium of the brain and transports DHA in the form of lysoPC or PC [see Lacombe et al. (2018) for extensive review]. Mfsd2a-knockout mice had markedly reduced levels of DHA in brain membranes, and this hallmark was accompanied by neuronal cell loss in the hippocampus and cerebellum as well as cognitive deficits, severe anxiety, and microcephaly, demonstrating the importance of that specific transporter in brain function (Nguyen et al., 2014; Zhou et al., 2019; Razmara et al., 2020; Wong and Silver, 2020). Mfsd2a deletion had major consequences on the development and proper functioning of the BBB (Ben-Zvi et al., 2014; Andreone et al., 2017). Interestingly, Mfsd2a also seems to be involved in the regulation of brain endothelial cell lipid composition, particularly in maintaining DHA levels, which may, in return, be unfavorable for caveolae formation and then contribute to the Mfsd2a-mediated regulation of transcytosis and barrier permeability (Andreone et al., 2017).

### Transport of Cholesterol Across the BBB

Unlike PUFAs, cholesterol in the CNS is almost entirely synthesized within the brain since the BBB prevents any direct transfer of sterols from the blood to the brain, especially when they are contained in lipoprotein particles (Dietschy and Turley, 2001; Björkhem et al., 2004). However, to compensate for the steady-state synthesis of cholesterol within the brain, there is a specific brain clearance mechanism. Cerebral microvessels have a certain level of ATP binding cassette protein A1 (ABCA1), a protein known to efflux cholesterol from the intracellular compartment to systemic and brain apolipoproteins. Do et al. (2011) reported that free cholesterol can be effluxed from the brain by crossing the BBB (Do et al., 2011). Therefore, the BBB might allow a certain level of free cholesterol to cross into the CNS, but one of the major limitations to this transfer is how cholesterol is transported within the blood, since the BBB seems to limit its uptake and more strongly favors its efflux from the CNS. Thus, the majority of cholesterol is directly synthesized in the brain parenchyma, mainly by glial cells and, to a lesser extent, by neurons (Mahley, 2016). Interestingly, dysfunction of or damage to the BBB led to altered cholesterol metabolism in the brain. Using a pericyte-deficient mouse model, Saeed et al. (2014) demonstrated that BBB disruption led to increased flux of cholesterol from the blood into the mouse brain and to a loss of 24(S)-hydroxycholesterol (the oxysterol regulating brain cholesterol synthesis) from the brain into the circulation, resulting in increased brain cholesterol synthesis.

In summary, a large proportion of lipids reach the CNS by crossing the BBB by using different strategies, including passive diffusion and specific and non-specific transporters. There is, however, another barrier where lipids can enter the CNS and reach the CSF, namely, the BCSFB, which is described in the following sections.

## Lipid Transport Across the Blood-CSF Barrier (BCSFB)

### Transport of Fatty Acids Across the BCSFB

So far, most of the studies performed on the transfer of PUFAs from the blood to the brain were performed at the BBB level. However, it is an open question whether PUFAs can enter the brain by crossing through the BCSFB. A way to address this question is to directly inject radioactive FAs into animals and test the *in vivo* metabolic activity of the CP. One study involved stereotaxic injection of radioactive palmitic acid in the left ventricle of anesthetized rabbits (Marinetti et al., 1971). One to eight hours after injecting the tracer, the animals were sacrificed, and the left, right, third, and fourth CPs were removed to determine how much radioactivity was recovered in the TG and PL classes in each CP. They reported that in the TG class, the level of the tracer peaked at 2 h post injection in the left ventricle and at approximately 5 h post injection in the fourth ventricle (Marinetti et al., 1971). With respect to PLs, the tracer peaked 4 h post injection, and the relative radioactivity of the tracer was higher in left ventricle PLs than in TGs. This group also evaluated the *in vitro* metabolic potential of each CP. After dissection, each CP was incubated in Krebs-Ringer buffer with radioactive palmitic acid for 1 h, and afterward, the lipids were extracted (Marinetti et al., 1971). Contrary to the *in vivo* study described above, the tracer was more highly incorporated into TGs than PLs in the *in vitro* study (Marinetti et al., 1971). The incorporation of the tracer was different among the four CPs, suggesting that the metabolism of palmitic acid is not the same in each CP and that each CP may have its own metabolism. The authors also suggested that CPs may use lipids as a source of energy for their metabolic functions, which is unlike white and gray matter. This is supported by the much greater enzymatic esterification of palmitic acid into TG in the CP than in white and gray matter (Marinetti et al., 1971). These data are in line with another study suggesting that TG accumulated in the CP when the PNPLA2 gene encoding adipose TG lipase (ATGL), which catalyzes the rate-limiting reaction of lipolysis, was knocked down in mice (Etschmaier et al., 2011). Another group tested whether the CP has delta-6 desaturase activity, as delta-6 desaturase is the key enzyme regulating chain elongation and desaturation of LA and ALA. They incubated CP with radioactive LA and evaluated the presence of radioactivity in the different lipid classes of the CP (Bourre et al., 1997). Approximately 50% of the radioactivity was recovered in the CP PCs, 20% in cholesteryl esters and 10% in phosphatidylethanolamine. An additional 2–5% of the radioactivity was detected in other PLs and in non-esterified FAs (Bourre et al., 1997). Taken together, these results suggest that CPs are involved in CNS PUFA metabolism and that there is a need to better understand their roles in brain PUFA homeostasis.

### Cholesterol Transport Through the BCSFB

**FIGURE 2:**
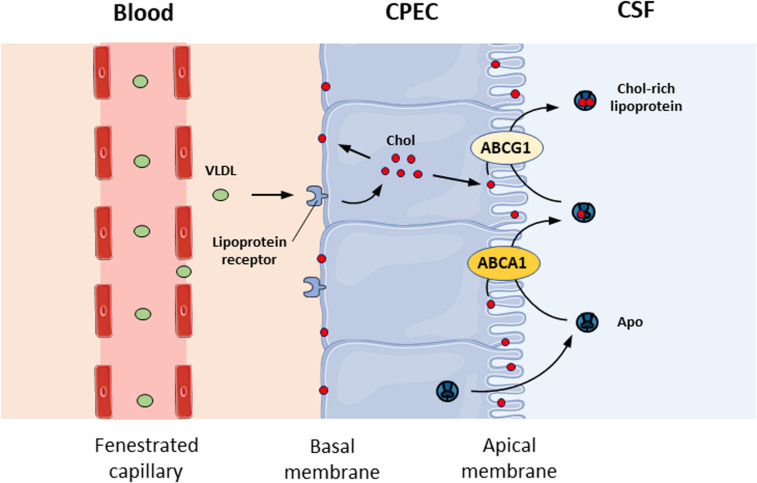
Cholesterol transport at the blood-CSF barrier. Schematic representation of cholesterol transport from the circulating blood to the CSF across the blood-CSF barrier. CPEC, choroid plexus epithelial cell; CSF, cerebrospinal fluid; Chol, cholesterol; VLDL, very low-density lipoprotein; Apo, apolipoprotein.

Cholesterol is essential for the structure and function of the CNS. It is the major constituent of myelin and plays a key role in synaptogenesis and neurotransmitter release (Mauch et al., 2001). The transfer of cholesterol to apolipoproteins and lipoproteins within the CSF plays an important role in brain homeostasis. Several genes involved in cholesterol metabolism are regulated by liver X receptors (LXRs) (Schultz et al., 2000). LXRs act as cholesterol sensors and modify the expression of genes that regulate the transport, catabolism, and elimination of cholesterol, such as cholesterol ester transfer protein, lipoprotein lipase, and apolipoprotein E (APOE) (Edwards et al., 2002). The mRNA expression of LXRα and LXRβ has been detected in rat CP (Fujiyoshi et al., 2007), suggesting that they could be important for the BCSFB. This is supported by the fact that CPs are barely detectable in LXRα^–/–^β^–/–^ mice because the size of lateral ventricles is greatly decreased, with little empty space left (Wang et al., 2002). The absence of LXRs in mice disrupts CNS lipid homeostasis since the accumulation of lipid droplets has also been observed within CPECs and around all four CPs of LXRα^–/–^β^–/–^ mice (Wang et al., 2002), suggesting a defect in cholesterol transport through the BCSFB. At a mechanistic level, several receptors, such as lipoprotein receptor-related protein 1 (LRP1), LRP2, and apolipoprotein E receptor 2 (apoER2), are expressed at the basal membrane of the CP epithelium (Kounnas et al., 1994; Kim et al., 1996) and involved in the uptake of very low-density lipoprotein (VLDL) from the circulating blood (Figure 2). The mechanisms by which cholesterol in the cytoplasm is integrated into the apical membrane of the CP are not known. CPECs have the capability to transfer cholesterol in the apical membrane to apolipoproteins and lipoproteins in the CSF with the help of ABCA1 and ABCG1, two membrane-bound transporters of the ABC transporter family that are expressed in the CP (Fujiyoshi et al., 2007). ABCA1 mediates the transfer of cholesterol but also PLs from cell membranes to either lipid-free or lipid-poor apolipoproteins such as apoA-I and apoE (Wang et al., 2000), while ABCG1 mediates cholesterol transfer to partially lipidated lipoproteins formed by the action of ABCA1 (Wang et al., 2004; Figure 1). Cholesterol release from CPECs occurs in an apical- and apoE isoform-dependent manner (Fujiyoshi et al., 2007).

The multiple functions of the CP include the release of biologically active molecules into the CSF by CPECs; these molecules are then distributed globally throughout the CNS (Lun et al., 2015a). This library of proteins consists of six major categories based on their biological functions, i.e., carrier proteins, matrix- or matrix-associated proteins, proteases, neurotrophic factors, and anti-inflammatory proteins (Thouvenot et al., 2006). It is possible that CPECs might coordinate cholesterol transport concomitantly with the release of apoE into the CSF. Interestingly, in ABCA1^–/–^ mice, the levels of apoE in the brain were reduced by 80% (Hirsch-Reinshagen et al., 2004), while apoA-I levels were dramatically increased in both the brain and CSF (Karasinska et al., 2009). These observations suggest that ABCA1 at the apical side of the CPECs also regulates the release of Apo proteins by the CP. Together, these studies show that the BCSFB plays an important role in regulating the transport of cholesterol from the peripheral blood circulation into the CSF. However, it is important to note that we currently do not know whether other mechanisms, such as the passive diffusion of FAs and the transcytosis pathway described for the BBB, take place in the BCSFB.

## How to Modulate Fatty Acid Membrane Composition

### At the BBB Level

BBB endothelial cells possess the metabolic capacity to synthesize ARA and DHA from their respective precursors (Moore, 2001). However, the synthesis of DHA from ALA or EPA, 20:5 is very limited. When ^14^C-labeled 22:5 n-3, 24:5 n-3, and 24:6 n-3 were used in endothelial cells, these intermediates were metabolized into DHA. The level of DHA synthesis from 22:5 n-3 was less than 1%, whereas the synthesis rate was higher when using 24:5 n-3 and 24:6 n-3 as precursors (Moore, 2001). According to Moore, the limiting step in the synthesis of DHA in endothelial cells involves the elongation of 22:5 n-3 into 24:5 n-3 and the desaturation of 24:5 n-6 into 24:6 n-3, the latter of which is catalyzed by Δ6 desaturase (Moore, 2001). In addition, several *in vitro* studies suggested that when 22:5 n-3 was supplied, the newly synthesized DHA was preferentially released into the culture medium, whereas the newly synthesized ARA was preferentially incorporated into the endothelial cell membranes (Benistant et al., 1995; Delton-Vandenbroucke et al., 1997). Numerous authors have thus suggested that BBB endothelial cells participate in the enrichment of DHA in the brain by supplying DHA directly to neurons (Delton-Vandenbroucke et al., 1997; Moore, 2001). In a study using coculture of endothelial cells and astrocytes, it was shown that these two cell types cooperated to provide more DHA to neurons (Moore, 2001). Endothelial cells in coculture actively converted over 50% of ALA into EPA, while astrocytes converted this precursor and, more specifically, EPA into DHA. This group also showed that more than 80% of synthesized n-3 PUFAs are not incorporated into the membrane PLs but are secreted into the culture medium. This longer-chain PUFA synthetic route would also be valid for the conversion of 18:2 n-6 to ARA. Therefore, it seems that there is metabolic cooperation between endothelial cells of the BBB and astrocytes to synthesize DHA and ARA and transport them into neurons (Moore, 2001). These results corroborate an *in vivo* study evaluating the FA composition of BBB endothelial cell membranes. Indeed, data obtained from animals fed a standard diet indicated that the brain microvessels of rats have a particularly high content of ARA compared to DHA (Selivonchick and Roots, 1977). In another study with rats fed a standard diet providing both LA and ALA, freshly isolated brain endothelial cells had higher levels of ARA and lower levels of DHA than the cortex, as evaluated in three PL classes [phosphatidylcholine (PC), ethanolamine phosphoglycerolipid (EPG), and phosphatidylserine (PS)] (Pifferi et al., 2005). In the microvessel PC fraction, saturated FAs accounted for 56% of the total fatty acids, and the ARA content was 60% lower than that in the cortex; DHA represented only 1.6% of the total fatty acids in the endothelial cell PC fraction, whereas it accounted for 7.6% of the cortex PC fraction. The capillary EPG fraction was ∼5 times richer in ARA than in DHA, whereas the ratio of these FAs was the opposite in the cortical EPGs (9.7% ARA and 22.5% DHA). ARA accounted for ∼4.5% of the total FAs in the PS fractions of microvessels and the cortex, but the PS DHA content was 84% lower in the microvessels than in the cortex. Thus, we concluded that the ARA:DHA ratios in the three PL classes were opposite in the microvessels and the cortex, confirming the specific enrichment of the brain parenchyma in n-3 fatty acids (Pifferi et al., 2005).

### At the BCSFB Level

To our knowledge, the first study reporting lipids in the CP was published by Helmy and Hack (1963). They evaluated the histochemistry and lipids of human CPs from individuals between 35 and 78 years of age. They performed thin-layer chromatography to separate the lipid classes but did not evaluate the FA profile; perhaps gas chromatography was not available at that time. Therefore, this study provided qualitative rather than quantitative data. They identified two phosphoinositides that seemed to be present in a greater relative proportion in the CP than in the CSF (Helmy and Hack, 1963). They also reported that unlike the CSF, the CP does not contain plasmalogens. Two decades later, Homayoun et al. (1988) reported the lipid and FA profile of CP. They performed FA profiling of the CP in rats fed for three generations with a diet with very low levels of ALA or with adequate levels of ALA. Regardless of the diet provided to the animals, Homayoun et al. (1988) reported that the CP was composed of 20% palmitic acid (16:0), 20% stearic acid (18:0), 10% oleic acid (18:1 n-9), and 25% ARA. When a diet low in n-3 PUFAs (low ALA, no DHA) was provided, CP had 25% higher behenic acid (22:0) and 60% lower DHA levels than rats fed adequate n-3 PUFAs (Homayoun et al., 1988). The CP of mice provided an ALA-deficient diet had 18% higher saturated FAs, 13% higher monounsaturated fatty acids (MUFAs), and 31% lower PUFAs than that of mice provided an adequate diet (Homayoun et al., 1988). There are similar modifications in the FA profile of brain capillaries during aging (Tayarani et al., 1989). Between 2 months and 24 months, the relative percentage of MUFAs in the brain capillaries of rats doubled, while that of PUFAs was reduced by half, suggesting that aging partially reproduces some of the outcomes of an n-3 PUFA-deficient diet (Tayarani et al., 1989). Although not statistically significant, one interesting change observed in the paper of Homayoun et al. (1988) was that the relative percentage of ARA was 35% lower in mice given the n-3 PUFA deficient diet than in mice given the adequate n-3 PUFA diet. This result is interesting since the relative percentage of ARA usually remains stable following dietary modification. In the paper of Tayarani et al. (1989), ARA in brain capillaries was reduced by more than half in 24-month-old rats compared to 2-month-old rats. Overall, the study of Homayoun et al. (1988) showed that providing a diet with very low levels of ALA shifted the FA profile to higher saturated and MUFA and lower PUFA; this is unusual since in brain membranes, the relative percentage of PUFA remains stable with lower % in n-3 PUFA shifting to a higher relative percentage of omega-6. They then investigated how long it would take to return the CP FA profile to “normal” by shifting the diet of animals fed the deficient diet to the “normal” n-3 PUFA diet. They concluded that 46 days of dietary treatment were required to reverse the n-3 PUFA-deficient profile of CP (Homayoun et al., 1988). It is therefore anticipated that these FAs are mostly incorporated into the PL bilayer of endothelial cells. It was a surprise to us that few studies have investigated the PUFA composition of the CP given the important functions of the CP-CSF system in brain homeostasis.

## Implications for Brain Lipid Metabolism and Effects on Health and Disease

Both the BBB and BCSFB undergo gradual modification during aging in which morphological and functional changes occur. Regarding the composition of brain lipids, between 20 and 100 years old, the brain will lose 42% in PL and 47% in cholesterol (Svennerholm et al., 1997). The effects of aging on the BBB have already been thoroughly documented [for review, see Mooradian (1988), Keaney and Campbell (2015), Erdö et al. (2017)]. These effects include (1) histological changes such as loss of capillary endothelial cells and decrease of their diameter, changes in endothelial cell morphology and decreased number of their mitochondria and (2) functional changes such as decreased BBB glucose and choline transport. Many studies have shown that diet and nutrition can improve cognitive functions during normal aging and can also prevent the development of Alzheimer’s disease (Ngandu et al., 2015; Rosenberg et al., 2018). However, for decades, researchers have not elucidated the DHA blood-to-brain link and its relation to cognitive scores. A recent meta-analysis of 11 cohort studies reported that higher DHA levels in blood were associated with better cognitive function in 22,887 individuals (van der Lee et al., 2018). However, whether this link exists because there is more DHA in the participants’ brain membranes remains unknown, largely due to the inaccessibility of brain samples and the quality of the samples collected at death to perform fatty acid profiling. We are aware of one study in humans that evaluated the link between plasma and postmortem brain DHA levels in non-cognitively impaired aged adults vs. cognitively impaired participants (Cunnane et al., 2012). The only significant correlation they obtained was between % DHA in plasma total lipids and % DHA in phosphatidylethanolamine of the angular gyrus, and this correlation was only observed in the non-cognitively impaired group. However, the brain requires a constant supply of DHA from the blood to replace metabolized DHA since astrocytic and neuronal synthesis of DHA from ALA is insufficient. Quantitatively, plasma non-esterified DHA (NE-DHA) is the main pool supplying the brain, whereas lyso-PC DHA is the most efficient, per dose, at targeting the brain (Chouinard-Watkins et al., 2018). Using *in situ* cerebral perfusion, brain DHA transport was studied in mice with targeted replacement knock-in of the human apolipoprotein E epsilon 4 allele (*hAPOE4*) since this is the main genetic risk factor for late onset Alzheimer’s disease. The brain uptake rate in *hAPOE4* mice perfused with NE-^14^C-DHA in the carotid was 24% lower than that in *hAPOE2* mice, in line with the reduced cortical DHA levels measured in *hAPOE4* mice during aging (Vandal et al., 2014). Conversely, in ∼35-year-old humans, a positron emission tomography study with [1-^11^C]-DHA reported that the mean global gray matter incorporation of DHA in the brain of *APOE4* carriers was 16% *higher* than that in non-carriers (Yassine et al., 2017), and this difference was particularly pronounced in the entorhinal region, an area affected early in AD pathogenesis, whereas the rate in the hippocampus was independent of the *APOE* genotype. This result means that when NE-DHA is available in the blood in a compartment that the brain can take up, the brains of young *E4* carriers can take up DHA, but the lyso-PC DHA pool might also be important in this population. In the 3x-TG transgenic mouse model of Alzheimer’s disease, the transport coefficient of DHA using *in situ* cerebral perfusion was 25% lower than that in non-transgenic littermates (Calon, 2011). Obviously, a reduction in the uptake of DHA through the BBB could lead to a deficit in DHA in the Alzheimer’s brain over the long term. Interestingly, this mouse model is vulnerable to a DHA-deficient diet since brain DHA levels were significantly lower than those in non-transgenic littermates after chronic exposure to the same deficient DHA diet as the transgenic model (Calon, 2011). There are evidence suggesting that mice with *hAPOE4* and transgenic models of Alzheimer’s disease might have issues in transferring plasma DHA to the brain membranes, and these issues might be accentuated during aging. Indeed, two studies reported a marked decrease in ARA in cerebral microvessels in aged rats compared with young rats (Tayarani et al., 1989; Bourre, 1991). Another group reported, however, that the FA composition of cerebral microvessels was unchanged between young and old rats, but they reported an increase in conjugated dienes, which could indicate increased free radical damage in old mice compared to young mice (Mooradian and Smith, 1992). A decrease in PUFAs within brain microvessel membranes will likely modify membrane fluidity. One of the potential consequences arising from such membrane FA modifications is the induction of membrane rigidity, which will impair the ability of the membrane to move to expose the active sites of receptors used to transport molecules across the BBB. Another lipid important to the brain membrane is cholesterol.

For the BCSFB, CPECs undergo physiological changes during aging, such as decreases in size, volume and microvilli length (Serot et al., 2003). Epithelial cells lose approximately 20% of their height, and their morphological shape flattens along with the shortening of their microvilli (Serot et al., 2003). Furthermore, the stroma becomes thicker, as do the walls of the blood vessels (Balusu et al., 2016). In addition, deterioration of the tight junctions, which act as a physical barrier in the BCSFB, has been observed during aging, and such barrier atrophy leads to a increased molecular trafficking and efflux into the CSF and the brain parenchyma (Balusu et al., 2016). These morphological changes in the CP also modify CSF secretion during aging; however, the release of apoE, one of the most highly expressed and secreted lipid carriers, does not exhibit any changes during aging (Silva-Vargas et al., 2016).

To our knowledge, there is no published data on the lipid transport and metabolism at the BCSFB during diseases. In the later stages of life, the CP-CSF axis shows a decline in all aspects of its functions, including protein synthesis and CSF secretion and turnover, which may themselves increase the risk of developing late-life diseases, such as AD. Both BBB and BCSFB modifications occurring during aging worsen in those with neurodegenerative diseases like AD (Damkier et al., 2013; Erdö et al., 2017). For instance, in patients with Alzheimer’s disease (AD), there is an additional loss to normal aging of 20% in PL in the frontal cortex (Svennerholm et al., 1997). In addition of the accumulation of beta-amyloid plaques and neurofibrillary tangles, one neurophysiopathological feature that has been underinvestigated by the research community is the presence of lipid droplets within the CNS. These hallmarks are also seen in non-AD patients, with a plateau at the age of 70 years (Marques et al., 2013), and correlate with observations made in aged mice (Sturrock, 1988). Although many efforts have been made to analyze and dissect the composition of CSF, it is important to emphasize that similar to plasma, CSF composition reflects the balance between the uptake and release of its metabolites between the CNS and peripheral circulation. Less effort has been made to investigate CP, the tissue that secretes CSF. Understanding the mechanisms that underlie the dysfunction of the CP-CSF system during aging will greatly advance the field. The brain utilizes a large amount of energy and requires tight regulation of nutrient intake and controlled management of the toxic molecules generated by its high catabolism. The regulation of brain input/output is closely tied to CSF turnover. CSF homeostasis is also important for maintaining proper brain structure and function. Given the importance of CSF turnover and composition, we believe that in-depth study of the human CP is now required.

## General Conclusion, Future Directions

In this review, we outlined some of the specific factors involved in the transport of long-chain PUFAs from the blood into the CNS. We found that there are a limited number of studies in this field, especially those pertaining to the BCSFB We believe that advancing the field requires the use of an advanced lipidomic approach, especially when studying the CP, which is a very small tissue. For instance, determining the lipidomic profiles of microvessels and CPECs would improve our knowledge of the physiological modifications of these two cell types that are central to the BCSFB. Such profiling could be performed in different species, such as rats, mice, primates, and humans, to appreciate the similarities and the differences among species. Modifications among species could provide important information as to whether lipid changes are involved in brain homeostasis mechanistic pathways or in the prevention of neurocognitive diseases. Our group has already shown that the metabolism of peripheral plasma n-3 PUFAs is modified during aging, which hence modifies the amount of n-3 PUFAs available for uptake by the aging brain. There are therefore many gaps in this field that could help prevent neurocognitive diseases that occur during aging, and new technologies, such as liquid chromatography coupled with mass spectrometry, are now well-equipped to make progress in this important and understudied field.

## Author Contributions

FP, BL, and MP wrote the manuscript, commented the manuscript, and contributed to improving the manuscript by providing comments. MP was responsible of the final version of the manuscript and its correspondence with the editorial board and the subsenquent comments this review may generate. All authors read the final version of the manuscript and agreed with its content.

## Conflict of Interest

The authors declare that the research was conducted in the absence of any commercial or financial relationships that could be construed as a potential conflict of interest.
